# Enhanced Cycleability of Amorphous MnO_2_ by Covering on α-MnO_2_ Needles in an Electrochemical Capacitor

**DOI:** 10.3390/ma10090988

**Published:** 2017-08-24

**Authors:** Quanbing Liu, Shan Ji, Juan Yang, Hui Wang, Bruno G. Pollet, Rongfang Wang

**Affiliations:** 1 School of Chemical Engineering and Light Industry, Guangdong University of Technology, Guangzhou 510006, China; liuqb@gdut.edu.cn; 2 College of Biological, Chemical Science and Chemical Engineering, Jiaxing University, Jiaxing 314001, China; 3 Institute of Chemical Engineering, Qingdao University of Science and Technology, Qingdao 266042, China; jing.kuang@firebright1.com (J.Y.); wanghui3931@126.com (H.W.); 4 Renewable Energy Group, Department of Energy and Process Engineering, Faculty of Engineering, Norwegian University of Science and Technology (NTNU), 7491 Trondheim, Norway; bruno.g.pollet@ntnu.no

**Keywords:** manganese dioxide, core-shell structure, porous, electrochemical capacitors, long stability

## Abstract

An allomorph MnO_2_@MnO_2_ core-shell nanostructure was developed via a two-step aqueous reaction method. The data analysis of Scanning Electron Microscopy, Transmission Electron Microscopy, X-Ray Diffraction and N_2_ adsorption-desorption isotherms experiments indicated that this unique architecture consisted of a porous layer of amorphous-MnO_2_ nano-sheets which were well grown onto the surface of α-MnO_2_ nano-needles. Cyclic voltammetry experiments revealed that the double-layer charging and Faradaic *pseudo*-capacity of the MnO_2_@MnO_2_ capacitor electrode contributed to a specific capacitance of 150.3 F·g^−1^ at a current density of 0.1 A·g^−1^. Long cycle life experiments on the as-prepared MnO_2_@MnO_2_ sample showed nearly a 99.3% retention after 5000 cycles at a current density of 2 A·g^−1^. This retention value was found to be significantly higher than those reported for amorphous MnO_2_-based capacitor electrodes. It was also found that the remarkable cycleability of the MnO_2_@MnO_2_ was due to the supporting role of α-MnO_2_ nano-needle core and the outer amorphous MnO_2_ layer.

## 1. Introduction

Electrochemical Capacitors (ECs) have been considered as promising electrochemical Energy Storage Devices (ESDs) due to their inherent advantages, such as high power density, long cycle life, high safety factor and environmentally benign nature [1]. With features complementary to batteries and fuel cells, ECs have been widely used as ESDs in many areas, such as in Smart Grids, Electric Vehicles (EVs) and Fuel Cell power systems [2]. Various transition metal oxides have been studied for ECs because of high capacitance originated of solid-state peseudofaradaic reaction, abundance and environmentally friendly properties. Among them, manganese dioxide (MnO_2_), a widely used cathode material in battery technologies, has been an attractive material in the area of ECs because of its advantageous properties [3,4,5,6,7]. In comparison to the multiple crystallographic MnO_2_ forms, amorphous MnO_2_ has been considered a predominant candidate as an EC electrode material due to its large initial capacity [8,9,10]. However, due to the poor cycleability of amorphous MnO_2_, this material has not been extensively used in ECs [11].

On the other hand, core-shell nanostructured MnO_2_@MnO_2_ can significantly improve the long cycling stability of MnO_2_, i.e., 90% retention after 20,000 cycles at a current density of 5 A·g^−1^. The authors found that the superior cycleability of the MnO_2_@MnO_2_ material was due to the core-shell nanostructure in which the wire-like ß-MnO_2_ core provided a stable structural backbone [12]. Similar findings were recently reported by Ma and co-workers who showed that the α-MnO_2_ nanowires acted as the stable backbone for δ-MnO_2_ nano-sheets to form a hierarchical structured composite which exhibited excellent cycling stability values, e.g., 98.1% retention after 10,000 charge-discharge cycles [13]. Furthermore, the cycling stabilities of both core-shell nanostructured MnO_2_@MnO_2_ electrodes are distinctly superior to those reported for other core-shell nanostructures composed of other transition metal oxides and MnO_2_ such as Co_3_O_4_@MnO_2_ [14,15,16], SnO_2_@MnO_2_ [17], NiO-MnO_2_ [18], and NiCo_2_O_4_/MnO_2_ [19].

Inspired and excited by the above findings in the literature, we produced a hierarchically allomorph α-MnO_2_@amorphous MnO_2_ core-shell structure, in which α-MnO_2_ needles were the core and amorphous MnO_2_ nano-sheets the shell. It was found that when the core-shell MnO_2_@MnO_2_ composite was used as an electrode, well-dispersed amorphous MnO_2_ layers on α-MnO_2_ needles allowed a fast, reversible Faradic reaction and enabled ion diffusion (due to the porous nature of the material). It was observed that a one-dimensional α-MnO_2_ needles acted as the backbone which maintained the structural integrity during the charge-discharge process, thus enhancing the cycling life of the shell. In addition, its porous feature of amorphous MnO_2_ (248.9 m^2^·g^−1^) also provided more contact area with the electrolyte, consequently the active surface was greatly improved. As expected, our designed α-MnO_2_@amorphous MnO_2_ hierarchical electrode exhibited a long-term stability during the cycling tests together with high specific capacitance and rate capability values.

## 2. Experimental

### 2.1. Synthesis of α-MnO_2_@amorphous MnO_2_

All chemical reagents were of high purity AR grade and used as received. The hierarchically core−shell α-MnO_2_@amorphous MnO_2_ nanostructure material was synthesized in two steps. In the first step, α-MnO_2_ needles were prepared by a hydrothermal reaction. Twenty milliliters of 8 mmol·L^−1^ KMnO_4_ solution was added into 20 mL of 4 mmol·L^−1^ MnSO_4_ solution with vigorous stirring. The above solution was topped up to 80 mL with ultra-pure water and then transferred to a 100 mL Teflon-lined autoclave, and heated at 140 °C for 12 h. Thereafter, the autoclave was allowed to cool to ambient temperature. The resulting solid product was collected by centrifugation, washed thoroughly with distilled water and then dried overnight in air at 60 °C. In the second step, the as-synthesized α-MnO_2_ needles were dispersed in 30 mL of 0.41 mmol·L^−1^ MnSO_4_ solution with vigorous stirring. Subsequently, 20 mL of 0.28 mmol·L^−1^ KMnO_4_ solution was added dropwise to the above solution. The reaction was maintained for 12 h. After that, the resulting solid product denoted as “MnO_2_@MnO_2_” was collected by centrifugation, washed thoroughly with distilled water and then dried overnight in air at 60 °C. For comparison purposes, amorphous MnO_2_ nano-sheets were also prepared in which the second-step procedure and materials were used but without the addition of the α-MnO_2_ needles.

### 2.2. Characterizations

X-ray diffraction (XRD) was carried out using a Shimadzu XD-3A (Shimadzu, Kyoto, Japan) fitted with a filtered Cu-Kα radiation (λ = 0.15418 nm) generated at 40 kV and 30 mA. The sorption isotherms were obtained on a Quantachrome Autosorb-1 volumetric analyzer. Specific surface area was determined by the Brunauer-Emmett-Teller (BET) method and the Density Functional Theory (DFT) was used for analyzing the full range of pore size distribution. Scanning Electron Microscopy (SEM) images were obtained on Carl Zeiss Ultra Plus (Carl Zeiss Microscopy GmbH, Jena, Germany). Fine structures were analyzed by Transmission Electron Microscopy (TEM) using a JEM-2010 (JEOL Ltd, Tokyo, Japan) microscope.

### 2.3. Electrochemical Measurements

Cyclic voltammograms (CV) and galvanostatic charge/discharge experiments were performed to evaluate the electrochemical behaviour of α-MnO_2_@amorphous MnO_2_ in a three-electrode cell configuration. The working electrode (WE) was fabricated by pasting a homogeneous slurry of α-MnO_2_@amorphous MnO_2_, carbon black and poly(tetrafluoroethylene) with a mass ratio of 80:10:10 into ethylene glycol, and then the mixture was rolled out to form uniform slices, followed by drying at 80 °C for 6 h. Subsequently, the slices were pressed onto a stainless steel (1 cm × 1 cm) plate with a tablet press. The amount of electrode material on the electrode was approximately 10 mg.

A three-electrode cell was used for the electrochemical experiments, in which the reference electrode (RE) was an Hg/HgO (1 M KOH) electrode and active carbon is working electrode (WE). The electrolyte was a 1.0 mol·L^−1^ LiOH. CV experiments were performed in the potential range (−0.14 V vs. Hg/HgO and +0.61 V vs. Hg/HgO) (0 to 0.75 V vs. SHE (standard hydrogen electrode)) at different scan rates of 2, 5, 10, 20 and 50 mV·s^−1^ on a CHI 650D electrochemical workstation. Galvanostatic charge/discharge (from −0.14 to 0.61 V vs. Hg/HgO) and cycle life tests were carried out using a Neware Battery Tester (Shengzhen Neware Technology Company, Shengzhen, China). Electrochemical Impedance Spectroscopy (EIS) analyses (Metrohm, Utrecht, The Netherlands) were performed between 10 kHz and 0.01 Hz using a 5 mV *RMS* sinusoidal modulation at the Open Circuit Potential (OCP).

## 3. Results and Discussion

In this study, α-MnO_2_ was synthesized in the first step by a hydrothermal process via the following reaction:

2KMnO_4_ + 3MnSO_4_ + 2H_2_O → 5MnO_2_ + K_2_SO_4_ + 2H_2_SO_4_ (1)

**Figure 1:**
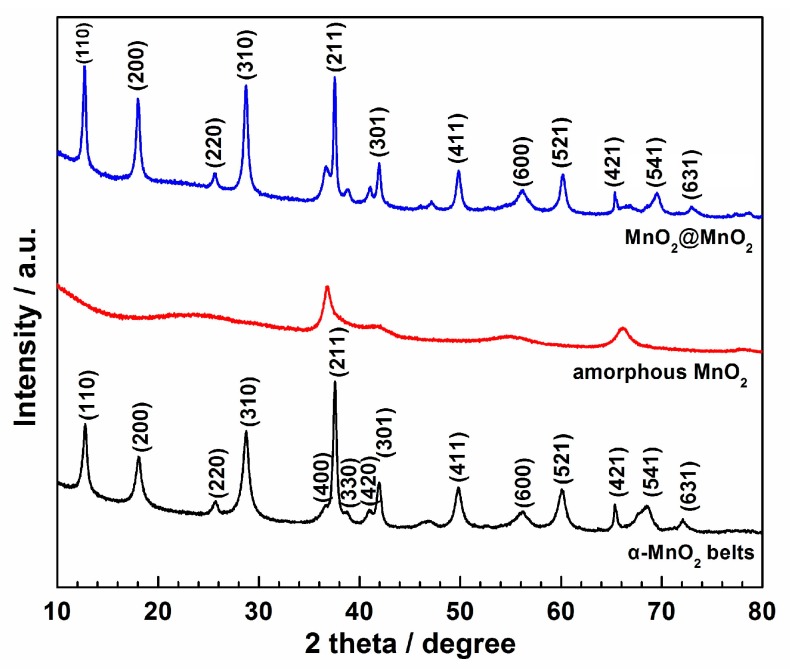
XRD patterns of MnO_2_@MnO_2_, α-MnO_2_ and amorphous MnO_2_ samples.

The XRD pattern of the material, shown as the black line in Figure 1, displays diffraction peaks characteristics for tetragonal α-MnO_2_ (JCPDS PDF 72-1982) [20]. The products obtained at the second step shown as the red line exhibit two broad diffraction peaks at around 37° and 66°, indicating the amorphous nature of the MnO_2_ sample [9]. When the amorphous MnO_2_ grew on the α-MnO_2_, the XRD pattern of MnO_2_@MnO_2_ (shown as the blue line) displays the characteristics of the two MnO_2_ samples, suggesting that the presence of the α-MnO_2_ base did not affect the structure of the amorphous MnO_2_.

**Figure 2:**
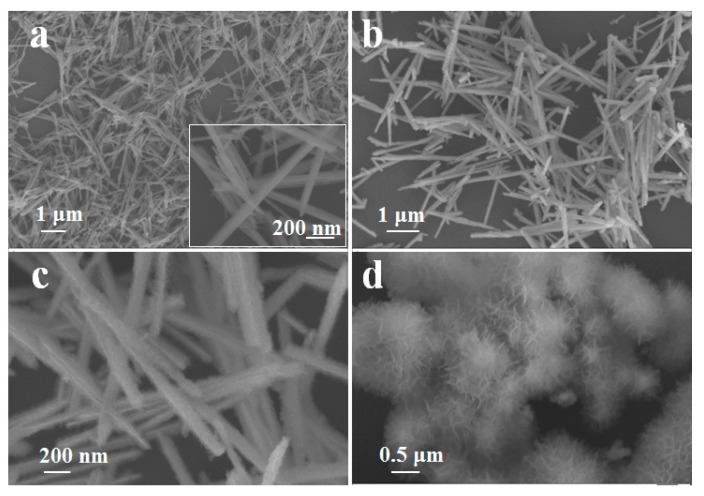
SEM images of: (**a**) α-MnO_2_ needles; (**b**,**c**) MnO_2_@MnO_2_; and (**d**) amorphous MnO_2_.

Figure 2 shows the morphologies of the three samples. It can be observed in Figure 2a that the as-prepared α-MnO_2_ needles have a width of 50–150 nm and a length extending from 1 to 5 μm. The inset of Figure 2a indicates that the needle-like α-MnO_2_ surface is smooth. The SEM image of MnO_2_@MnO_2_ shown in Figure 2b reveals that the length of the needles was kept unchanged. The high resolution SEM image in Figure 2c clearly shows a cotton-like surface, resulting from the uniform growth of amorphous MnO_2_. Meanwhile, no packed amorphous MnO_2_ was observed in the space among the needles, indicating that the amorphous MnO_2_ preferred to cover on the surface of the α-MnO_2_ needles. When the α-MnO_2_ needles were not present, the amorphous MnO_2_ would aggregate into pompom-like clusters.

**Figure 3:**
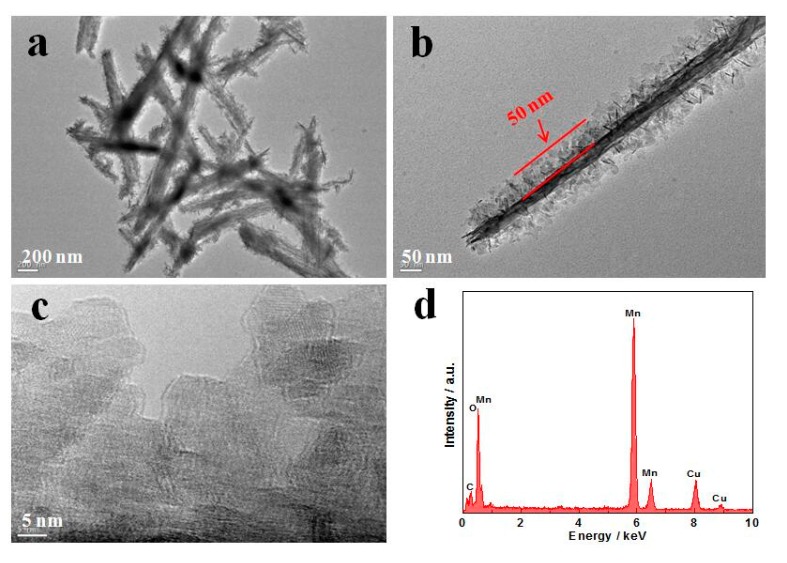
(**a**–**c**) TEM images with different magnification; and (**d**) EDX (Energy-dispersive X-ray) spectrum of MnO_2_@MnO_2_.

Further structural characterizations of the MnO_2_@MnO_2_ were carried out by TEM. Figure 3a shows a typical TEM image of the MnO_2_@MnO_2_, revealing that the hierarchically nanostructure is constructed by numerous thin nano-sheet “shells” covered on the needle “core”, which is in very good agreement with the SEM observation. From Figure 3b, it was possible to determine a shell thickness of ca. 50 nm. The figure also shows that the shell layer had an open structure with the interconnected nano-sheets creating abundant space. High resolution TEM of the shell in Figure 3c did not display clear lattice fringe, indicating poor crystallinity of the shell, which is in agreement with the XRD results. The EDX pattern, shown in Figure 3d, indicates that MnO_2_@MnO_2_ consisted principally of the elements of manganese and oxygen.

**Figure 4:**
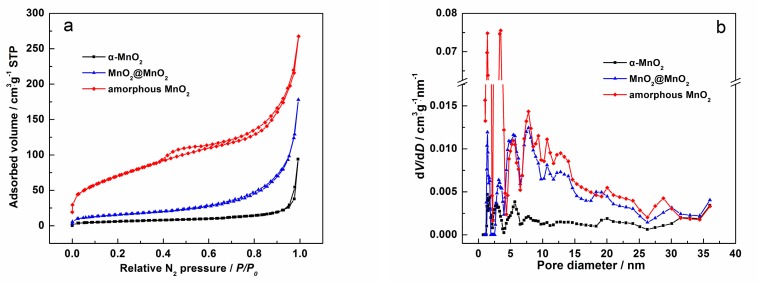
N_2_ adsorption-desorption isotherms (**a**) and the pore size distribution (**b**) of α-MnO_2_, MnO_2_@MnO_2_ and amorphous MnO_2_.

N_2_ adsorption-desorption isotherms of α-MnO_2_, MnO_2_@MnO_2_ and amorphous MnO_2_ are shown in Figure 4. From the figure, it can be observed that α-MnO_2_ needles displayed *type II* isotherms according to the IUPAC classification. The isotherms of amorphous MnO_2_ could be classified as *type IV* [21]. A distinct hysteresis loop can also be observed in the larger range of 0.4–1.0 *P*/*P*_0_ indicating a relatively large pore size [22]. When composite α-MnO_2_ needles and amorphous MnO_2_ were both present, *type III* isotherms were observed for MnO_2_@MnO_2_, which could be the mix of *type II* and *IV* isotherms. Their pore size distributions are shown in Figure 4b. As observed, the three samples had micro- and mesopores; and the amount of the pores increased following the sequence of α-MnO_2_ < MnO_2_@MnO_2_ < amorphous MnO_2_. The BET specific surface areas of α-MnO_2_, MnO_2_@MnO_2_ and amorphous MnO_2_ were found to be 21.2, 54.4 and 248.9 m^2^·g^−1^, respectively. The total pore volume values calculated from the total nitrogen adsorption were 0.15, 0.28 and 0.41 cm^3^·g^−1^, respectively. The relatively low BET surface area and total pore volume of MnO_2_@MnO_2_ compared to amorphous MnO_2_ can be attributed to the presence of α-MnO_2_ in the hierarchical structure.

**Figure 5:**
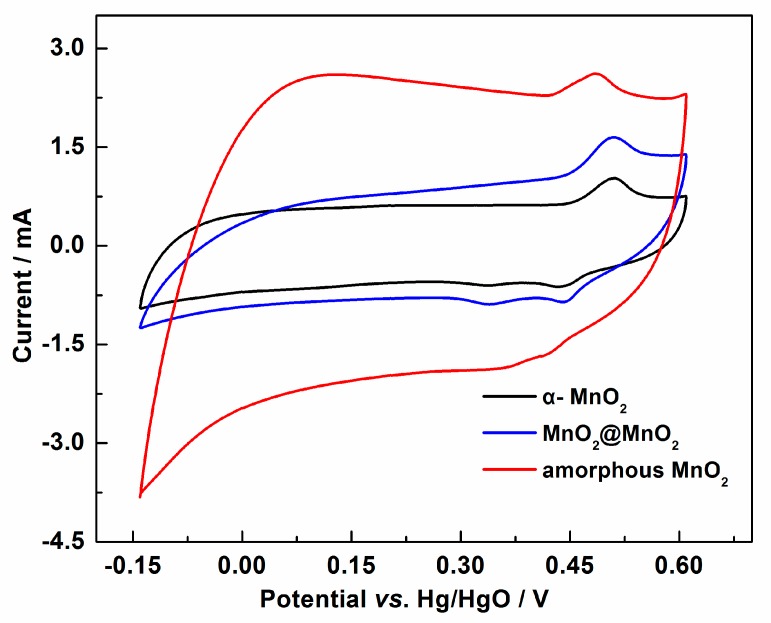
CV curves of the α-MnO_2_, MnO_2_@MnO_2_ and amorphous MnO_2_ electrodes at scan rate of 5 mV·s^−1^.

Electrochemical performance of MnO_2_@MnO_2_ was firstly studied by Cyclic Voltammetry (CV) in the potential window of (−0.14; +0.61 V (vs. Hg/HgO)). Figure 5 shows cyclic voltammograms recorded at a scan rate of 5 mV·s^−1^. The figure also shows that all CV curves are “roughly” of symmetric rectangular shapes, typical of *pseudo*-capacitive behavior, and the redox peak potentials of +0.49 V and +0.44 V vs. Hg/HgO correspond to the reversible Faradaic redox reaction, indicating that the electrode was charged and discharged through the intercalation/deintercalation of Li^+^ into the MnO_2_ structure accompanied by the valence conversion of Mn^4+^ to Mn^3+^ [23]. This finding also indicates that the capacitance of the three capacitor electrodes consists of the double-layer charging and Faradaic *pseudo*-capacity. It was found that the voltammetric current response decreased in the following sequence: amorphous MnO_2_ > MnO_2_@MnO_2_ > α-MnO_2_, indicating that the capacitance decreased in a similar trend, which could be attributed to a decrease of the BET surface area, leading to a decrease of the double-layer charging.

**Figure 6:**
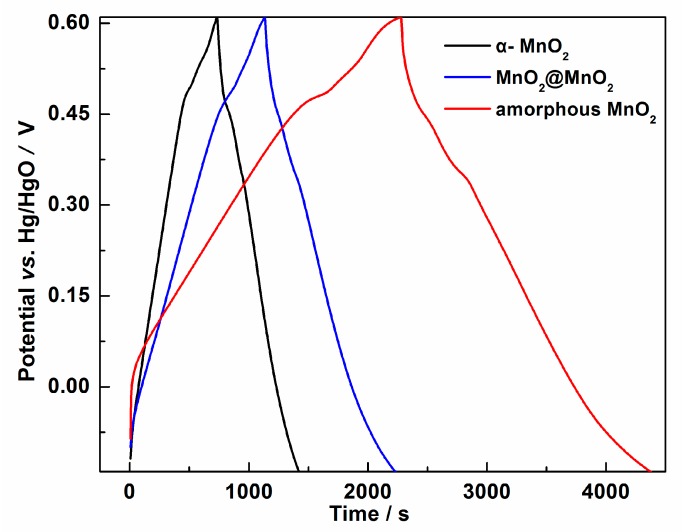
Charge-discharge behavior of the α-MnO_2_, MnO_2_@MnO_2_ and amorphous MnO_2_ electrodes at current density of 0.1 A·g^−1^.

To further investigate the cyclability of MnO_2_@MnO_2_, galvanostatic charge/discharge experiments were performed at constant current densities of 0.1 A·g^−1^ as shown in Figure 6. The figure shows that the three samples exhibited linear variation in potential during the charging/discharging process and had *quasi-*symmetrical shapes, indicating a clear contribution from the *pseudo*-capacitive and double layer processes [24]. Based upon the galvanostatic discharge curves, the specific capacitance was calculated according to the following Equation (2):

*C* = *I*Δ*t*/*m*Δ*V* (2)

where *C* (F·g^−1^) is the specific capacitance, *I* (mA) is the charge-discharge current, Δ*t* (s) is the discharge time, *m* (mg) represents the mass of the electrode (active material), and Δ*V* (V) is the potential drop during discharge. The specific capacitance of the α-MnO_2_, MnO_2_@MnO_2_ and amorphous MnO_2_ electrodes were calculated to be 91.2, 150.3 and 247.0 F·g^−1^ respectively. From the data, it can be observed that amorphous MnO_2_ exhibited the highest specific capacitances, which resulted from the largest surface area among the three samples. This observation is in excellent agreement with other reported findings which state that the use of amorphous MnO_2_ as EC electrode material exhibits large initial capacity compared to the multiple crystallographic MnO_2_ forms, as shown in Table 1 [8,9,10]. It was also found that the specific capacitances of the as-prepared MnO_2_@MnO_2_ were larger than isomorphous MnO_2_@MnO_2_ electrode (108 F·g^−1^ at 5 A·g^−1^) [12], but smaller than the allomorph MnO_2_@MnO_2_ electrode (310.2 F·g^−1^ at 0.5 A·g^−1^) [13]. In addition, it was also observed that the presence of α-MnO_2_ is not favorable for the initial capacity of amorphous MnO_2_.

**Figure 7:**
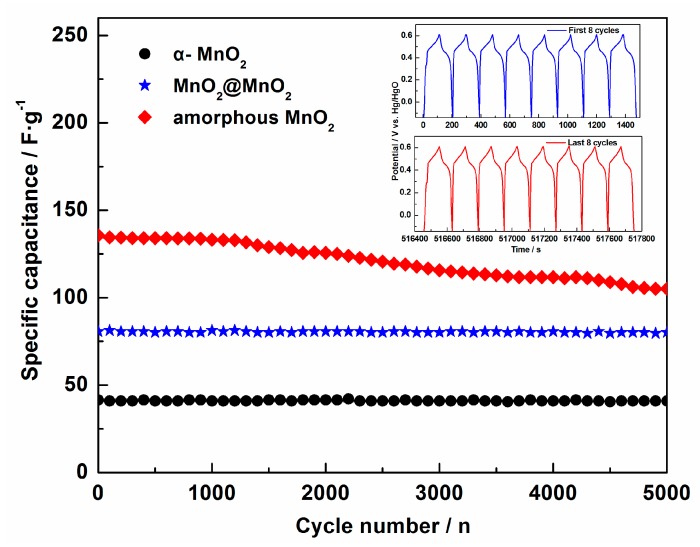
Cyclic stability of the three electrodes in a 1 mol·L^−1^ LiOH electrolyte measured using the galvanostatic charge-discharge technique at a current density of 2 A·g^−1^.

To evaluate the cyclic stability of the MnO_2_@MnO_2_ electrode, galvanostatic charge/discharge tests were performed at a current density of 2 A·g^−1^. As shown in Figure 7, after 5000 cycles, the specific capacitance retention of the α-MnO_2_, MnO_2_@MnO_2_ and amorphous MnO_2_ electrode remained 99.1%, 99.3% and 77.4% of their initial capacity respectively, indicating a good cyclic stability for α-MnO_2_ and MnO_2_@MnO_2_. As seen in the inset, the comparison of the charge-discharge curves of MnO_2_@MnO_2_ between the first (1st) and the eighth (8th) cycles indicates that the curves are very stable, further emphasizing the long-term stability of MnO_2_@MnO_2_. In addition, it was found that the retention of MnO_2_@MnO_2_ after 5000 cycles was higher than that of many MnO_2_-based composite electrodes [14,26,27,28,29]. This finding clearly demonstrates that the presence of α-MnO_2_ needles can efficiently improve the cycleability of amorphous MnO_2_.

**Figure 8:**
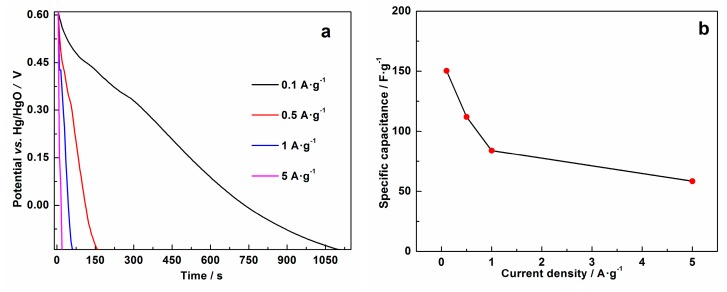
(**a**) Galvanostatic charge-discharge curves of the MnO_2_@MnO_2_ electrode at different current densities of 0.1, 0.5, 1 and 5 A·g^−1^, respectively; and (**b**) variation in specific capacitance with current density obtained based on Figure 8a.

Rate capability testing of MnO_2_@MnO_2_ was evaluated at current densities ranging from 0.1 A·g^−1^ to 5 A·g^−1^. Figure 8 shows that the specific capacitance decreased as the current density increased, standard finding which is in good agreement with reported literature on MnO_2_-based hybrids. From 0.1 A·g^−1^ to 5 A·g^−1^, the specific capacitances of MnO_2_@MnO_2_ decreased from 150.3 F·g^−1^ to 58.4 F·g^−1^, corresponding to a 38.9% retention of its initial capacitance. The decreasing trend of the capacitance indicates that some parts of the MnO_2_@MnO_2_ structure are inaccessible for the intercalation of Li^+^ ions at high current density. Compared to some reported MnO_2_-based capacitor electrodes [5,30], the rate capability of as-prepared MnO_2_@MnO_2_ is comparable, which could be ascribed to its unique structure, namely the outer porous structure act as a continuous pathway for the diffusion of electrolyte, which can shorten solid state transport distances for ions into the MnO_2_ structure.

**Figure 9:**
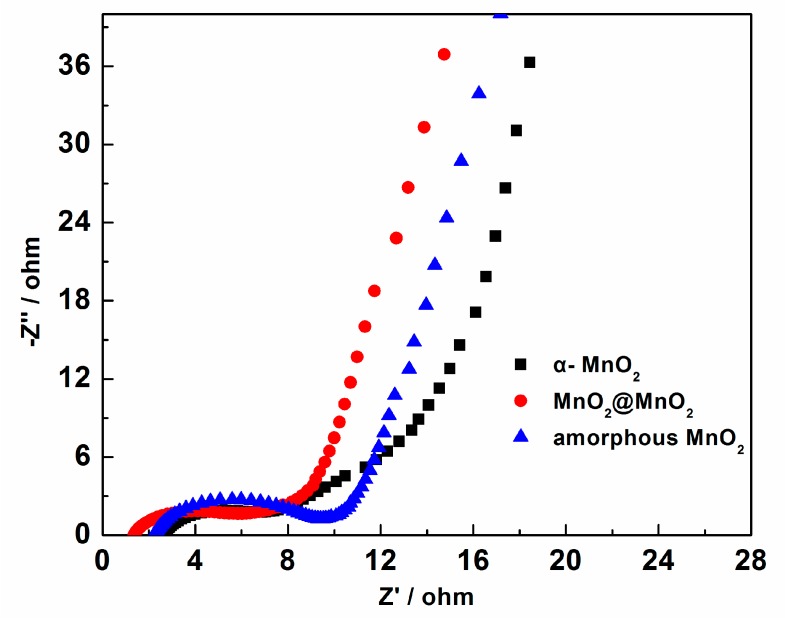
*Nyquist* plots of the electrochemical impedance spectroscopy for the three electrodes.

To elucidate the causes behind the difference in capacitance among the three electrodes, EIS experiments were performed. *Nyquist* plots of the three electrodes were generated. Figure 9 displays: (i) semicircles located at the high frequency region which could be related to the charge transfer process at the electrode/electrolyte interface; and (ii) straight lines at the low frequency region possibly ascribed to the ion diffusion process in the bulk of the active mass. It can also be seen in Figure 9 that the semicircles decrease in the sequence of: amorphous MnO_2_, MnO_2_@MnO_2_, and α-MnO_2_, suggesting that the charge transfer resistance decreases from amorphous MnO_2_ to α-MnO_2_. It may be observed that the linear slope of amorphous MnO_2_ is larger than that of MnO_2_@MnO_2_ and α-MnO_2_, suggesting that the ion diffusion resistance for amorphous MnO_2_ is lower than MnO_2_@MnO_2_ and α-MnO_2_. These results indicate that the charge transfer resistance of amorphous MnO_2_ can lead to improved capacitor by mixing with α-MnO_2_ needles, but the ion diffusion resistance is increased, which could be attributed to the large different of the porous structure between the amorphous MnO_2_ and the α-MnO_2_ needles.

## 4. Conclusions

A hierarchical α-MnO_2_ nano-needles@amorphous MnO_2_ thin nano-sheet core-shell nanostructure was fabricated by a two-step aqueous reaction method. As an electrode for *pseudo*-capacitors, at a current density of 0.1 A·g^−1^, the constructed MnO_2_@MnO_2_ exhibited a promising specific capacitance of 150.3 F·g^−1^, which consisted of the double-layer charging and Faradaic *pseudo*-capacity. Importantly, nearly 99.3% retention after 5000 cycles at a current density of 2 A·g^−1^ was found. Such intriguing electrochemical properties may be attributed to the hierarchically allomorphs core-shell configuration, in which 1*D* MnO_2_ nano-needle core acted as a stable structural backbone and 2*D* amorphous MnO_2_ provided a large surface area and more active sites. Thus, the fabricated MnO_2_@MnO_2_ electrode material with a unique hierarchically allomorphs core-shell architecture are promising candidate material for high-cycleability in Energy Storage Devices.

## Figures and Tables

**Table 1:** Comparison of the specific capacitance of as-prepared electrodes to other reported amorphous MnO_2_ and MnO_2_@MnO_2_ electrodes.

Samples	[Ref.]	The specific Capacitance (F·g^−1^)	Electrolyte Type & Concentration	Scan Rate or Current Density
Amorphous MnO_2_	herein	247	1.0 M LiOH	0.1 A·g^−1^
MnO_2_@MnO_2_	herein	150	1.0 M LiOH	0.1 A·g^−1^
Amorphous MnO_2_	[8]	242	0.5 M Na_2_SO_4_	2 mA·cm^−2^
Amorphous MnO_2_	[9]	110	2 M NaCl	5 mV·s^−1^
Amorphous MnO_2_	[10]	225	1.0 M Li_2_SO_4_	2 mV·s^−1^
		242	1.0 M Na_2_SO_4_	
		194	0.5 M K_2_SO_4_	
Amorphous MnO_2_	[25]	79	1.0 M Na_2_SO_4_	1 A·g^−1^
MnO_2_@MnO_2_	[12]	108	0.5 M Na_2_SO_4_	5 A·g^−1^
MnO_2_@MnO_2_	[13]	310	6.0 M KOH	0.5 A·g^−1^
